# A Narrative Review of the Evolution of Diagnostic Techniques and Treatment Strategies for Acral Lentiginous Melanoma

**DOI:** 10.3390/ijms251910414

**Published:** 2024-09-27

**Authors:** Myoung Eun Choi, Eun Ji Choi, Joon Min Jung, Woo Jin Lee, Yoon-Seo Jo, Chong Hyun Won

**Affiliations:** Department of Dermatology, Asan Medical Center, University of Ulsan College of Medicine, Seoul 05505, Republic of Korea; mechoi316@naver.com (M.E.C.); dpsk702@gmail.com (E.J.C.); bban29@hanmail.net (J.M.J.); uucm79@hanmail.net (W.J.L.); yoon-seo.jo@nhs.net (Y.-S.J.)

**Keywords:** acral lentiginous melanoma, dermoscopy, pathogenesis, preferentially expressed antigen in melanoma, prognosis, tumor microenvironment, surgery

## Abstract

Acral melanoma (AM) is a subtype of cutaneous melanoma located on the palms, soles, and nails. The pathogenesis of AM involves mechanical stimulation and characteristic tumor-promoting mutations, such as those in the *KIT* proto-oncogene. Dermoscopy is useful for diagnosing AM, which is characterized by parallel ridge patterns and irregular diffuse pigmentation. Although histopathological confirmation is the gold standard for diagnosing AM, lesions showing minimal histopathological changes should be considered early-stage AM if they clinically resemble it. Recently, immunohistochemical staining of preferentially expressed antigen in melanoma has been recognized as a useful method to distinguish benign from malignant melanocytic tumors. Research reveals that AM is associated with an immunosuppressive microenvironment characterized by increased numbers of M2 macrophages and regulatory T cells, alongside a decreased number of tumor-infiltrating lymphocytes. Mohs micrographic surgery or digit-sparing wide local excision has been explored to improve quality of life and replace wide local excision or proximal amputation. AM has a worse prognosis than other subtypes, even in the early stages, indicating its inherent aggressiveness.

## 1. Introduction

Acral melanoma (AM) is a distinct subtype of cutaneous melanoma (CM) that develops on the palms, soles, and nails. Subungual melanoma (SUM) is an anatomical subtype of AM that arises from the nail matrix.

AM differs from other subtypes of CM due to its unique ethnic predilection, pathogenesis, and prognosis. Although the overall incidence of CM is significantly higher in individuals with lighter skin, AM constitutes a larger proportion of CM in darker-skinned populations [1,2,3]. A study using the Surveillance, Epidemiology, and End Results (SEER) registry from 2006 to 2015 found that the age-adjusted incidence rate of AM was 2.0 per million person-years, with no significant differences among racial groups. However, the proportion of AM was highest in Black individuals (33.6%), followed by Asian/Pacific Islanders (23.1%), Hispanic whites (9.3%), and non-Hispanic whites (1%) [4]. Furthermore, AM has a poorer prognosis in terms of overall survival and disease-specific survival compared with other types of CM. According to the SEER database, the 5-year melanoma-specific survival rate for AM was 80.6%, significantly lower than the overall rate for CM, which was 93% [4]. Similarly, a study using the National Cancer Database reported a 5-year overall survival rate of 67.3% for AM, compared with 75.8% for non-acral CM [5]. The 5-year overall survival rates for AM in other countries vary widely, ranging from approximately 50% to 80% [3,6,7,8,9,10]. It is believed that the poor prognosis of AM is primarily due to more advanced tumor thickness and stage at the time of diagnosis [11]. In some studies, AM continued to show a poorer prognosis even after adjusting for Breslow depth and stage [12,13]. The worse survival outcomes may be attributed to differences in the tissue microenvironment between acral and non-acral skin, such as the exclusive expression of *KRT9* in acral sites (associated with melanocyte function and proliferation) and decreased expression of *CCL27* (a chemokine that attracts T cells to the skin). Additionally, differences in genomic drivers and immune cell infiltration (increased numbers of M2 macrophages and lower numbers of tumor-infiltrating lymphocytes) may also play a role [14,15,16,17].

In this article, we discuss the current knowledge regarding the pathogenesis, diagnosis, and management of AM, with a focus on recent advancements in these areas.

## 2. Methods

We conducted a PubMed search using the terms “acral” and “melanoma”, which yielded 647 results. We then limited the publication dates to 1990–2024 and excluded articles not in English, narrowing the results to 587. Given the broad scope of the disease, a systematic review was not feasible. Therefore, we prioritized meta-analyses, systematic reviews, randomized clinical trials, and original articles that provided new insights and demonstrated the highest study quality.

## 3. Pathogenesis

AM is believed to have a distinct pathogenesis compared with other subtypes of CM due to its unique characteristics. It often occurs in weight-bearing areas, suggesting that mechanical stimulation plays a role in its development [3]. In a retrospective study involving 685 patients with CM conducted at two medical centers in China, the extremities exhibited a higher risk of post-trauma melanoma compared with other sites [18]. Minagawa et al. [19] retrospectively identified the sites of 123 plantar AMs and found a predilection for both the rear and front of the foot, consistent with a study involving 177 Korean patients with CM that indicated a higher incidence in physically stressed areas, such as the inner forefoot and heel [3]. These studies support the hypothesis that shear stress may promote the evolution of AM. However, the exact mechanism by which trauma induces melanoma development remains largely unknown. One study suggested that traumatic events or chronic wounds could lead to local inflammation, with interactions between neutrophils and pre-neoplastic cells resulting in the proliferation of these cells [20]. Additionally, weight-bearing activities could activate YAP, which may decrease nuclear envelope integrity in cells undergoing tumorigenesis [21]. Sustained mechanical stress could contribute to the rupture of nuclear and micronuclear membranes in malignant melanoma cells, leading to genomic instability [21].

Additionally, AM commonly affects non-sun-exposed areas, suggesting that ultraviolet (UV) radiation does not play a significant role in its development, unlike other subtypes of CM. Supporting this idea, recent studies of whole-genome sequences in melanomas have shown that AM exhibits fewer UV signature mutations compared with non-acral CM [22,23]. Instead of UV signature mutations, AM has higher numbers of structural variants and focal copy number variants but lower numbers of single-nucleotide variants and small insertions/deletions (indels) [22].

Moreover, AM has characteristic tumor-promoting mutations that are distinct from those found in other subtypes of CM. Mutations in the BRAF proto-oncogene (*BRAF*) and the NRAS proto-oncogene (*NRAS*) occur at lower rates in AM compared with other types of CM [22,24,25]. Conversely, mutations in neurofibromin 1 (*NF1*) and the KIT proto-oncogene (*KIT*), along with oncogenic amplifications of genes such as *CCND1*, *PAK1*, *GAB2*, *CDK4*, and telomerase (*TERT*), are more common in AM than in CM [22,24,25,26].

Recently, Belote et al. [27] proposed that subpopulations of melanocytes exhibit anatomical site-specific enrichment during fetal development, which persists into adulthood. In their study, melanocytes were classified into two subtypes based on gene expression: volar-like (v-mel) and non-volar cutaneous-like (c-mel). Although both v-mel and c-mel cells were found across various anatomical sites, v-mel cells were particularly enriched in volar skin [27]. The transcriptional signature of the volar-enriched population, or v-mel cells, was retained in AM, suggesting that AM originates from a specific subset of melanocytes, thereby differing inherently from other types of CM. This finding may help explain the poorer prognosis of AM compared with other CM subtypes [27].

Very recently, Liu et al. revealed that the invasive AM subset is characterized by subclonal diversification, increased epithelial–mesenchymal transition, and spatial enrichment of APOE^+^/CD163^+^ macrophages [28]. In vitro and ex vivo experiments further demonstrated that APOE^+^/CD163^+^ macrophages promote tumor epithelial–mesenchymal transition via insulin-like growth factor 1 (IGF1)–IGF1 receptor interactions, suggesting critical molecular biomarkers for AM [28].

## 4. Diagnosis

**Figure 1:**
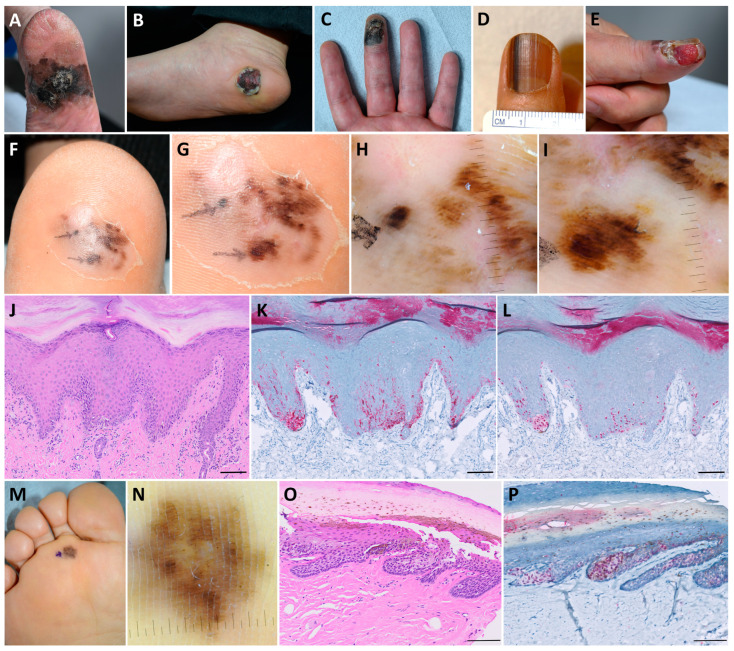
Clinical, histopathological, and immunohistochemical features of acral melanoma. (**A**–**C**) Clinical features of acral melanoma on the soles and palm. Clinical features of subungual melanoma presenting as melanonychia (**D**) and amelanotic nodule (**E**). (**F**,**G**) Acral melanoma showing asymmetry, border irregularity, multiple colors, and diameter > 6 mm. (**H**,**I**) Dermoscopic findings of acral melanoma showing parallel ridge patterns and irregular diffuse pigmentation. (**J**) Skin biopsy reveals non-equidistant single melanocytes. Malignant melanocytes are positive to HMB-45 (**K**) and PRAME (**L**) staining. (**M**,**N**) Clinical and dermoscopic features of early acral melanoma showing mild asymmetry and multiple colors. (**O**) Irregular and confluent nests are observed in skin biopsy (**P**) Melanocytes are positive to PRAME staining (scale bar = 100 μm; (**J**) H&E, (**K**) HMB-45, (**L**) PRAME, (**O**) H7E, and (**P**) PRAME; ×400).

Given the research findings indicating that AM has a poor prognosis due to late diagnosis, it is crucial to identify early cases of AM to improve outcomes. However, early AM and SUM may appear similar to benign nevi and melanonychia, respectively, making early diagnosis challenging. The diagnosis of AM and SUM relies on clinical, dermoscopic, and molecular features (Figure 1A–E). Herein, we discuss relevant findings related to these conditions.

### 4.1. Clinical Findings

Because the standard “ABCDE” rule (asymmetry, border irregularity, color, diameter > 6 mm, and evolution) is not well-suited for evaluating AMs, the acronym “CUBED” has been proposed to highlight clinical features that raise concern for foot malignant melanoma [29]. “CUBED” stands for colored lesions, uncertain diagnosis, bleeding lesions, enlargement or deterioration of a lesion, and delayed healing beyond 2 months. However, this acronym lacks specific morphological criteria, and there have been no further studies to confirm its clinical utility in AM [30].

The differential diagnosis for AM includes a broad spectrum of pigmented diseases, such as congenital and acquired acral nevi, lentigo, and trauma-related hemorrhage [31]. The hypopigmented or amelanotic type of AM is often confused with infectious lesions, pyogenic granulomas, plantar warts, poromas, porocarcinomas, and squamous cell carcinoma [32].

### 4.2. Dermoscopic Findings

Dermoscopy is a valuable adjunctive tool for diagnosing AM (Figure 1F–I,M,N). Two common dermoscopic patterns observed in AM are parallel ridge patterns and irregular diffuse pigmentation [33,34]. The former is considered the most potent predictor for AM, whereas the latter is primarily associated with invasive AM [35]. Saida et al. [33] reported the sensitivity and specificity of the parallel ridge pattern for AM as 86.4% and 99.0%, respectively, and for irregular diffuse pigmentation as 85.4% and 96.6%, respectively. However, some benign lesions can also exhibit these patterns; therefore, other clinical clues should be incorporated to differentiate benign lesions from AM [36,37].

The revised 3-step algorithm and the BRAAFF algorithm are two well-known approaches for managing acquired melanocytic lesions affecting acral volar skin [35,38]. The three steps of the 3-step algorithm are as follows: first, check for a parallel ridge pattern; second, identify any typical dermoscopic patterns of benign acral nevus throughout the lesion (such as parallel furrow, lattice-like, or fibrillar patterns); and third, assess whether the lesion’s maximum diameter exceeds 7 mm [35]. The BRAAFF checklist includes the following criteria: irregular blotch, parallel ridge pattern, asymmetry of structures, asymmetry of colors, parallel furrow pattern, and fibrillar pattern [38]. This checklist differs from the 3-step algorithm in that it includes assessments for blotches and asymmetry while excluding lesion size considerations. The BRAAFF algorithm has demonstrated 93.1% sensitivity and 86.7% specificity for diagnosing AM [38].

However, in cases of very early-stage melanoma in situ, dermoscopic features can be subtle and may be confused with lentiginous nevi. A recent study examining dermoscopic findings in AM according to lesion size revealed that small AMs (<15 mm) are less likely to exhibit typical patterns, such as the parallel ridge pattern (54.5%), irregular diffuse pigmentation (27.3%), and gray color (18.2%), compared with larger AMs (≥15 mm) [37]. The authors concluded that close follow-up is necessary for atypical, small evolving melanocytic lesions.

### 4.3. Histopathological Findings

Although histopathological confirmation is essential for diagnosing AM, differentiating between AM and other benign lesions, especially nevi, can be challenging. Architectural and cytological features must be carefully evaluated to accurately distinguish malignant from benign conditions.

In terms of architectural characteristics, malignant lesions are typically composed of solitary tumors that coalesce into nests. These nests are often non-cohesive, poorly circumscribed, confluent, and vary in size [30] (Figure 1J,O). Additionally, malignant lesions exhibit non-equidistant single units, whereas benign lesions display uniform growth of melanocytes [30]. In AM, melanocytes often ascend along the crests, corresponding to the dermoscopic “parallel ridge pattern” [39].

In early-stage AM, architectural features may be subtle, and cellular atypia can provide crucial diagnostic clues [40]. Melanoma should be suspected if multiple features are present, including melanocytic nuclei more than twice the size of keratinocytic nuclei, vertical arrangement of melanocytic nuclei, angulation of nuclei, thick or elongated dendrites reaching the upper parts of the epidermis, or highly hyperchromatic and prominent nucleoli [40,41,42]. However, it is important to note that even lesions with minimal histopathological changes should be considered a precursor phase of AM if they clinically mimic malignancy [43]. Previously, these lesions were diagnosed as atypical melanosis of the foot with uncertain malignant potential [44]. Many authors now argue that these lesions represent an early developmental phase of AM as they exhibit a high percentage of cyclin D1 gene amplifications and dermoscopic features indicative of malignancy, such as the parallel ridge pattern and irregular diffuse pigmentation [43,45]. Therefore, when pigmented lesions meet the clinical criteria for AM, they should be managed as an early phase of AM, even in the absence of identifiable histopathological changes, as they may represent an indolent subtype of AM with a prolonged radial growth phase [43,46,47].

### 4.4. Immunohistochemical (IHC) Staining

IHC staining can be valuable in differentiating AM from nevi. Several IHC markers, such as S-100, HMB-45, and MART-1/Melan-A, are used to distinguish AM from benign melanocytic nevi. HMB-45 is highly specific for melanoma cells (Figure 1K), whereas S-100 is a highly sensitive marker [48,49]. A previous study reported the sensitivities of S-100, HMB-45, and MART-1/Melan-A for AM as 95%, 80%, and 70%, respectively [50]. However, no combination of antibodies allows for unequivocal differentiation between melanoma and melanocytic nevi [51]. Recently, IHC staining of preferentially expressed antigen in melanoma (PRAME), a tumor-associated antigen, was widely explored to distinguish between benign and malignant melanocytic tumors. Lezcano et al. [52] reported diffuse expression of PRAME in 94.4% (17/18) of AMs, including in situ tumors, whereas 86.4% (121/140) of melanocytic nevi showed completely negative staining. Among benign lesions, only one pigmented junctional Spitz nevus exhibited diffuse staining [52].

In another study specifically examining PRAME in AM, 65.3% (49/75) of cases showed diffuse staining [53] (Figure 1L,P). When the cutoff for distinguishing AM from acral nevi was set at staining at least 50% of lesional melanocytes, the sensitivity and specificity of PRAME were 69.3% and 100%, respectively [53]. Further elaborating on the value of PRAME in diagnosing melanoma, Lezcano et al. [54] analyzed 110 diagnostically problematic melanocytic tumors, comparing the results from PRAME IHC with those from fluorescence in situ hybridization (FISH) and/or single nucleotide polymorphism array, alongside the final diagnostic interpretation. PRAME IHC staining and cytogenetic test results showed concordance in 90% of cases, and 92.7% of cases demonstrated agreement between PRAME IHC staining and the final diagnosis [55]. However, PRAME expression may be less sensitive or specific depending on melanoma subtypes, and interpreting weak immunoreactivity can be challenging [56]. The limitations of PRAME IHC are well-summarized in the review by Lezcano et al. [56].

In summary, the aforementioned studies suggest that IHC, including PRAME, can provide additional support for the diagnosis of AM.

### 4.5. FISH

FISH is being investigated as a diagnostic tool for AM. Su et al. [57] studied the sensitivity and specificity of 3-probe and 4-probe FISH assays. The 3-probe FISH assay targeted 8q24 (*MYC*), 9p21 (*CDKN2A*), and *CEP9* (centromere 9), whereas the 4-probe FISH assay targeted 6p25 (*RREB1*), *CEP6* (centromere 6), 6q23 (*MYB*), and 11q13 (*CCND1*). Among 44 AM cases, the sensitivity was 70.5% for the 4-probe FISH assay and 59.1% for the 3-probe FISH assay [57]. However, when both probe sets were combined, sensitivity increased to 88.6%. Specificity was 100%, as none of the 36 benign acral nevi showed gene alterations [57].

Lai et al. [58] tested 51 dysplastic nevi and 58 melanoma cases using a 4-color FISH assay. Among the melanoma cases, 37 were in situ, 22 were classified as Clark level 2, and 42 were acral [58]. The sensitivity of the 4-color FISH assay in differentiating between the two groups was 94.9%, with a specificity of 94.0% [58]. In a proposed algorithm by Darmawan et al. for managing acral melanocytic lesions, the 4-probe FISH assay was incorporated into the workflow for assessing melanocytic lesions with ambiguous biopsy features [30].

For accurate and early diagnosis of AM, clinicians should integrate clinical, dermoscopic, and molecular features.

## 5. Tumor Microenvironment

The tumor microenvironment is a complex ecosystem composed of various cell types, including immune cells, fibroblasts, endothelial cells, and the extracellular matrix. A fundamental component of this environment is tumor-infiltrating lymphocytes (TILs), a subpopulation of lymphocytes that demonstrate enhanced immunological reactivity against tumor cells. A study suggested that TILs are associated with prognosis and response to immune checkpoint inhibitors [59].

Although there is some controversy, several studies have indicated that AM exhibits lower levels of TILs compared with non-AM, which correlates with a diminished response to immune checkpoint inhibitors [60,61,62]. Additionally, Zúñiga-Castillo et al. [17] demonstrated significantly increased infiltration of M2 macrophages, an immunosuppressive subtype linked to melanoma aggressiveness and progression, in both peritumoral and intratumoral regions of AM compared with superficial spreading melanoma. Recent advancements in single-cell RNA sequencing have revealed that AM has a significantly lower number of natural killer cells, plasmacytoid dendritic cells, CD8 T cells, and γδ T cells compared with non-AM [63]. Another single-cell analysis highlighted a higher abundance of regulatory T cells and exhausted CD8 T cells [64]. Although heterogeneity exists within the tumor microenvironment of AMs, most studies indicate that AM is associated with a more immunosuppressive tumor microenvironment compared with other types of melanomas. Furthermore, IHC staining studies have shown that AM has a lower frequency of PD-L1 expression compared with other melanoma subtypes [65].

## 6. Sentinel Lymph Node Biopsy (SLNB)

The National Comprehensive Cancer Network (NCCN) guidelines generally recommend considering SLNB for T1b melanoma, as the expected positivity rate of sentinel lymph nodes (SLNs) is 5–10% [66]. For T2a-T4b melanoma, the NCCN suggests discussing and offering SLNB, given that the likelihood of a positive SLN exceeds 10% [66]. Although the NCCN guidelines do not differentiate among cutaneous melanoma subtypes in terms of SLNB utility, previous studies have indicated that AM has a higher SLN positivity rate than non-acral CM [67,68]. An analysis of the SEER database from 1998 to 2013 revealed an SLN positivity rate of 25.7% for AM across various stages [67]. A recent study involving 959 patients with AM from the National Cancer Database found that the AM subtype was independently associated with the highest risk for SLN positivity [68].

## 7. Surgical Resection

The standard surgical approach for cutaneous melanomas involves wide local excisions with margins determined based on Breslow thickness, as recommended by the NCCN. Mohs micrographic surgery is recognized in the NCCN guidelines for selected sites, including acral sites [66,69]. However, assessing melanocytic atypia in frozen sections can be limited; thus, the additional use of IHC staining, such as MART-1 and SOX-10, can aid in interpreting margin status [69,70,71,72]. Slow Mohs micrographic surgery, which utilizes formalin-fixed paraffin-embedded sections for margin assessment, is a more time-consuming procedure that may result in prolonged open wounds [73]. Nevertheless, it ensures complete tumor clearance and maximizes the conservation of acral tissue, preserving digit function even in invasive cases [71]. A recent study demonstrated that slow Mohs micrographic surgery was superior to wide local excision in terms of recurrence rates in AM [73]. Additionally, it reduced postoperative defect size and shortened wound-healing time in AM [73].

Traditionally, AMs located on the digits were managed through digital amputation at the metacarpal–interphalangeal joint to optimize survival [74]. However, the significant functional impairment caused by amputation has led to an evolution in surgical approaches, shifting from proximal amputation to partial digit amputation, Mohs micrographic surgery, and digit-sparing wide local excision [69,75]. Proponents of digit-sparing techniques argue that these methods may not compromise survival while improving quality of life [76,77,78,79,80,81]. However, due to the limited number of studies and the retrospective nature of existing research, clear guidelines for the application of digit-preserving surgery have yet to be established.

## 8. Systematic Therapy for Advanced AM

Despite lower levels of TILs and a reduced frequency of PD-L1 expression, recent advancements in immunotherapy have shown that anti-PD-1 and anti-CTLA-4 combination therapies are beneficial for patients with AM and are now considered the standard of care [82,83,84]. A large-scale international study by Bhave et al. found a significantly higher overall response rate of 43% in the anti-PD-1/anti-CTLA-4 combination group, compared with 26% for the anti-PD-1 monotherapy group and 15% for the anti-CTLA-4 monotherapy group [85]. In their study, patients with *BRAF* mutations demonstrated better responses to all types of immune checkpoint inhibitors [85]. Similarly, a large-scale study from Japan reported that combination therapy was associated with a higher overall response rate compared with monotherapy (40% vs. 16%) [82]. Notably, this study indicated that SUM responded significantly better to combination therapy than to monotherapy (overall response rates of 61% vs. 10%), a trend not observed in patients with non-nail unit AM [82]. Combination therapy was linked to a higher incidence of treatment-related adverse events compared with anti-PD-1 monotherapy, including diarrhea (44.1% vs. 19.2%), rash (40.3% vs. 25.9%), and pruritus (33.2% vs. 18.8%) [86].

Patients with AM receiving anti-PD-1 monotherapy had higher overall survival rates at 12 months (53%) compared with those on anti-CTLA-4 monotherapy (34%; *p* < 0.001), as indicated by a meta-analysis [87]. However, studies have shown that patients with AM generally exhibit lower response rates and shorter durations of response compared with those with other subtypes of CM [63,87]. In general, overall response rates, progression-free survival, and median overall survival with immunotherapy were found to be lower in studies conducted in China and Japan compared with those based in the U.S. or Europe [85,88,89,90,91,92]. To date, reliable systemic treatment guidelines for AM have not yet been established.

Furthermore, due to the lower rate of somatic mutations in AM compared with non-acral CM, targeted therapies play a limited role in the treatment of AM [93]. The response rates of BRAF and BRAF-MEK inhibitors in BRAF-mutant AM are similar to those observed in BRAF-mutant non-acral CM [94]. Conversely, a systematic review investigating the efficacy of c-KIT inhibitors found a pooled overall response rate of 22% for AM and 14% for overall CM [95]. Among c-KIT inhibitors, imatinib demonstrated a slightly higher overall response rate (27%) than nilotinib (22%) in patients with AM [95].

## 9. Prognosis

AM is reported to have a poorer prognosis compared with other melanoma subtypes. This is often attributed to delays in diagnosis, which can result in deeper Breslow thickness, a higher frequency of ulceration, greater lymph node involvement, and an increased rate of distant metastases [12,96,97]. The independent prognostic significance of the acral lentiginous subtype has been debated [98,99,100]. Recently, a study involving a large cohort of T1 thin melanomas—comprising 129 acral lentiginous melanomas and 699 other histological subtypes—found that the acral lentiginous subtype was associated with lower disease-specific survival, overall survival, and recurrence-free survival, as well as a higher rate of sentinel lymph node metastasis [101]. Consistent with these findings, a multicenter study conducted across three Italian and one Polish melanoma referral centers, which included stage I–II melanomas (4349 superficial spreading melanomas, 2132 nodular melanomas, and 253 acral lentiginous melanomas), indicated that the acral lentiginous subtype was significantly associated with worse disease-free survival in multivariable analysis [102]. These studies suggest that acral lentiginous melanomas exhibit inherent aggressiveness even in the early stages [101,102].

**Figure 2:**
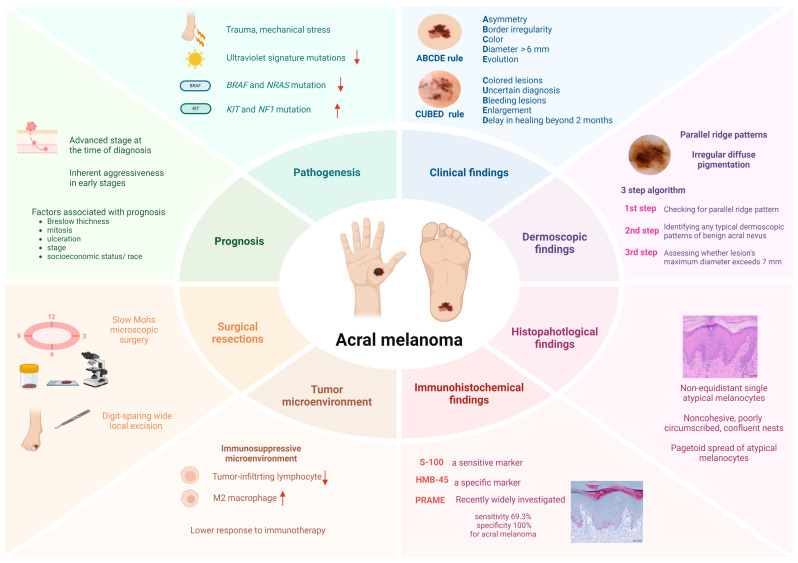
Summary of acral melanoma. Acral melanoma is associated with higher rates of *KIT* and *NF-1* mutations. The general “ABCDE” rule and the “CUBED” rule are used for clinical assessment. Common dermoscopic patterns include the parallel ridge pattern and irregular diffuse pigmentation. Diagnosis relies on histopathological and immunohistochemical findings, with PRAME immunohistochemical staining being a recent area of investigation. Acral melanoma is characterized by an immunosuppressive microenvironment, and surgical approaches have evolved to include slow Mohs microscopic surgery and digit-sparing wide local excision, enhancing quality of life. Overall, the prognosis of acral melanoma is poorer than that of other melanoma subtypes. (Upward pointing arrow in this figure means increased while downward pointing arrow means decreased).

In addition to established prognostic factors for cutaneous melanomas, such as Breslow thickness, mitotic rate, ulceration, and stage, survival outcomes for AM also vary by race [12]. A recent study utilizing a specialized SEER registry in the United States examined the impact of socioeconomic status and race on AM survival outcomes [103]. The hazard ratios for Hispanic, Black, and Asian/Pacific Islander patients were found to be 1.48, 1.25, and 1.1, respectively, compared with non-Hispanic White patients in fully adjusted Cox regression models [103]. Furthermore, this study suggests that socioeconomic status partially contributes to increased mortality among Hispanic and Black patients [103]. The overall features of AM are summarized in Figure 2.

## 10. SUM

SUM, a subtype of AM, exhibits unique genetic features and prognosis compared with other subtypes. Although SUM is classified as AM, studies have indicated that it has distinct genomic profiles compared with AM [24,104]. SUM displays a higher mutation burden and more UV radiation mutational signatures than AM at other sites, suggesting that the nail apparatus provides insufficient protection from UV radiation. Additionally, SUM has a relatively higher incidence of *KIT* and *NF-1* mutations while showing a lower incidence of *BRAF* and *NRAS* mutations compared with AM at other sites [105].

Because SUM most frequently presents as melanonychia, the differential diagnosis of longitudinal pigmentation is crucial [106]. Melanonychia can arise from non-melanocytic origins, melanocytic activation, or melanocytic proliferation [107]. Non-melanocytic pigmentation includes conditions such as fungal melanonychia and subungual hemorrhage [107]. In fungal melanonychia, longitudinal pigmentation is often associated with dermatophyte infections, whereas diffuse brown pigmentation is typically linked to black molds [108]. Melanonychia caused by melanocytic activation may result from trauma, periungual tumors, medications, systemic diseases, or nail apparatus lentigo [107]. Conversely, melanonychia related to melanocytic proliferation includes nail matrix nevi and SUM [107]. Nail matrix nevi are more commonly found in children than in adults, and childhood nail matrix nevi tend to be darker, multicolored, more triangular, and exhibit more dots or globules, often displaying a pseudo-Hutchinson’s sign [109].

For the diagnosis of SUM, clinical, dermoscopic, and molecular findings are utilized, similar to AM. The “ABCDEF” rule is commonly employed to raise awareness for SUM, which stands for age (20–90 years, peaking in the 5th–7th decades), band (brown to black pigment, wider than 3 mm, or with irregular/blurred borders), change (rapid size growth or lack of improvement in nail dystrophy despite treatment), digit involved (thumb > hallux > index finger; single digit > multiple digits or dominant hand), extension of pigment (beyond the nail plate, indicating Hutchinson’s sign), and family history (of previous melanoma or dysplastic nevus) [110]. However, Ko et al. [111] found no significant difference in the number of ABCDEF criteria met between the SUM group and the benign longitudinal melanonychia group among patients who underwent biopsy, suggesting that the number of criteria met is not a reliable indicator of malignancy [111].

Many conditions can present as melanonychia, making early detection of SUM reliant on differentiating it from benign conditions [112]. Malignant melanocyte proliferation or SUM should be suspected when melanonychia is monodactyl, multicolored, and rapidly enlarging in adults. Onychoscopy may reveal a brown background with longitudinal lines that are irregular in color, width, spacing, and parallelism [113,114]. However, Di Chiacchio et al. found that the accuracy of nail plate dermoscopy for diagnosing melanoma in situ of the nail matrix was only 53% among experts, indicating that onychoscopy serves as a supplementary diagnostic tool [115]. Additionally, a recent study identified that factors such as color intensity, variegation, band change, and Hutchinson’s sign, along with nail plate splitting, were independently associated with SUM compared with benign melanonychia [116].

In nail matrix biopsies, SUM should be suspected if there are >30 melanocytes per 1 mm length of the epidermal/dermal junction. Cytological features such as nuclear enlargement, nuclear atypia, hyperchromatism, irregular contours of nuclei, and intraepithelial mitoses may also indicate malignancy [40,117]. A study performing 3-probe and 4-color FISH on 23 cases, including 18 in situ SUM and five thin SUM, found that 65.2% (15/23) of cases were positive [117]. Consistent with previously reported findings in AM, *CCND1* again demonstrated the highest proportion of positive cases [117].

**Figure 3:**
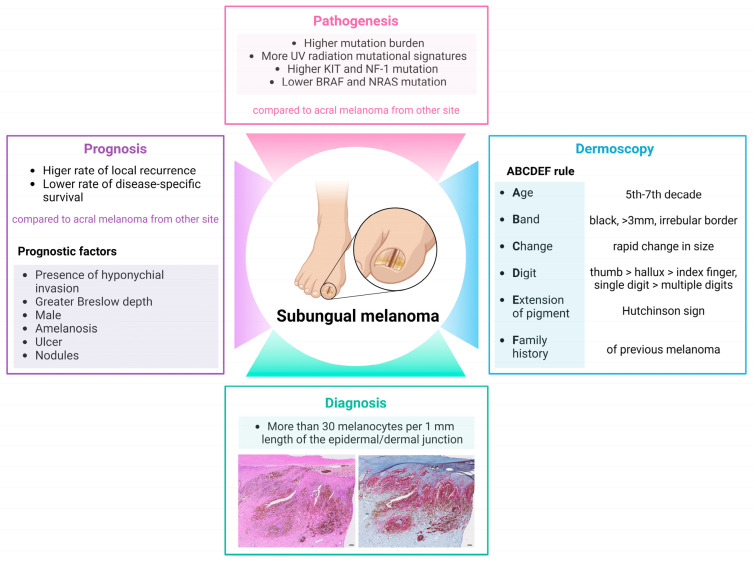
Summary of subungual melanoma. Subungual melanoma has a distinct pathogenesis compared with acral melanoma at other sites. The “ABCDEF” rule is utilized to raise awareness for subungual melanoma. Diagnosis is based on histopathological findings, and the prognosis of subungual melanoma is worse than that of acral melanoma at other sites.

Several studies have indicated that the prognosis of SUM is worse than that of AM at other sites [118,119] A study evaluating survival data based on the location of AM revealed that SUM was associated with a higher tendency for local recurrence compared with AM at other sites [118]. Additionally, research suggests that disease-specific survival rates are significantly lower in SUM than in AM at other sites [119]. In terms of prognosis and recurrence after treatment, the presence of hyponychial invasion and Breslow thickness have been investigated. Yoo et al. found that hyponychial invasion was significantly associated with Breslow depth, metastasis, and disease-free survival among anatomical subunits [120]. Factors such as male sex, amelanotic color, ulcers, and nodules, along with greater Breslow depth, were associated with a higher risk of recurrence in SUM [121]. A Breslow depth shallower than 0.8 mm was linked to a better indication for functional surgery rather than amputation [121]. Moreover, studies have shown that a history of trauma in patients with SUM is an independent risk factor for worse survival outcomes [105,122]. Further research is needed to confirm these findings and elucidate the mechanisms behind the poorer prognosis of SUM. The characteristics of SUM are summarized in Figure 3.

## 11. Conclusions

Although AM is a subtype of melanoma, it exhibits distinct characteristics that differentiate it from other CM subtypes. Due to its location on the non-glabrous skin of acral sites, the development of AM is linked to mechanical stress, and it shows fewer UV signature mutations [21,22]. Diagnosing AM can be challenging, as it is often identified at later stages compared with other melanoma subtypes [99]. The standard “ABCDE” and “CUBED” rules serve as useful guides for identifying clinical features that warrant further evaluation. Familiarity with common dermoscopic patterns, such as parallel ridge patterns and irregular diffuse pigmentation, can significantly enhance the diagnosis of AM [29,35]. The final diagnosis relies on histopathological features, which typically reveal an increased number of non-equidistant malignant cells or poorly circumscribed, confluent nests [30]. However, caution is necessary, as the early developmental phase of AM may lack identifiable histopathological changes in skin biopsies, risking the oversight of an indolent subtype [43,46]. In differentiating AM from benign pigmented lesions, IHC markers such as S-100, HMB-45, MART-1/Melan-A, and PRAME can be valuable tools [48,53].

In spite of the controversies, several studies have indicated that AM is associated with an immunosuppressive microenvironment characterized by fewer TILs and natural killer cells, more M2 macrophages, and lower PD-L1 expression in melanoma cells [68,87]. This environment may correlate with a higher sentinel lymph node positivity rate and a lower response rate to immunotherapy in AM compared with non-acral CM [17,63,64,65]. Despite these findings, combination therapies with anti-PD-1 and anti-CTLA-4 have shown promise for patients with AM [82,83]. Surgical approaches for local AM have evolved from proximal amputation to slow Mohs micrographic surgery and digit-sparing wide local excision, aiming to improve the quality of life [69,75]. Prognostically, AM continues to demonstrate poorer survival outcomes even after adjusting for stage, suggesting inherent biological aggressiveness [101,102].

SUM, a subtype of AM, exhibits distinct genetic features and prognosis. As SUM often presents as melanonychia, it is crucial to differentiate longitudinal nail pigmentation using the “ABCDEF” rule for SUM and onychoscopy [106,110]. Although further research is needed, existing studies indicate that the survival outcomes for SUM are worse than those for AM at other sites [118,119].
